# Delta family protocadherins contribute to protoglomerular targeting of olfactory sensory neuron axons in the olfactory bulb

**DOI:** 10.1371/journal.pgen.1012090

**Published:** 2026-04-01

**Authors:** Daniel T. Barnes, Ezekiel M. D. Crenshaw, Matthew J. Curran, Jessica B. Herr, Emily S. Devereaux, Carly D. Seligman, Jonathan A. Raper

**Affiliations:** 1 Department of Biology, University of Pennsylvania, Philadelphia, Pennsylvania, United States of America; 2 Department of Neurosciences, Perelman School of Medicine, University of Pennsylvania, Philadelphia, Pennsylvania, United States of America; 3 Department of Natural & Applied Sciences, Cheyney University, Cheyney, Pennsylvania, United States of America; Cleveland Clinic Cole Eye Institute, UNITED STATES OF AMERICA

## Abstract

To understand how neural circuits are assembled, it is essential to identify and characterize the axonal guidance cues and receptors that determine the axonal trajectories and connections between neurons. We performed single-cell RNA sequencing of olfactory sensory neurons from zebrafish to identify candidate axonal guidance-related genes that are differentially expressed according to sensory axon target location in the olfactory bulb. Among the candidates we identified were several members of the non-clustered delta-protocadherin family of adhesion molecules. We found that two members of the delta1-protocadherin family, *pcdh7b* and *pcdh11*, are most highly expressed in sensory neurons that project to a specific identifiable neuropil in the early olfactory bulb called the DZ protoglomerulus. Knocking down either one of these protocadherins impairs the ability of sensory axons to terminate within the DZ protoglomerulus. Knockdown does not affect the ability of other sensory axons from terminating normally in a separate neuropil called the CZ protoglomerulus. In contrast, two members of the delta2-protocadherin family, *pcdh10b* and *pcdh17*, are most highly expressed in sensory neurons that project to the CZ protoglomerulus. Knocking down *pcdh10b* induces ectopic terminations of CZ projecting sensory axons. Knocking down *pcdh17* induces substantial ectopic axonal trajectories and impairs CZ projecting sensory axons from finding and terminating in the CZ protoglomerulus. Knockdowns of either *pcdh10b* or *pcdh17* do not affect DZ projecting sensory axons. We conclude that delta1-protocadherins help DZ projecting sensory axons enter and remain within the DZ protoglomerulus, while delta2-protocadherins help CZ projecting sensory axons navigate to the CZ protoglomerulus.

## Author summary

There are more than 100 odorant receptor (OR) genes in the Zebrafish genome, and each olfactory sensory neuron expresses only one or two very closely related ORs early in their development. As sensory neurons extend axons into the olfactory bulb of the brain, they seek out and terminate in specific, reproducibly located neuropils that are related to the OR they express. This occurs before these axons further segregate into smaller, OR-specific neuropils. Overall, these observations suggest that OR choice and sensory axon guidance are closely coordinated. We identified a family of adhesion molecules, the non-clustered delta-protocadherins, as potential axon guidance-related cues based on their differential expression in olfactory sensory neurons targeting different locations in the bulb. Knocking each of these candidate cues down demonstrates their functional contribution to olfactory axon targeting in specific subsets of sensory neurons. Delta1-protocadherins contribute to the targeting of sensory axons to one neuropil region while delta2-protocadherins contribute to the targeting of sensory axons to another. These findings identify non-clustered protocadherins as contributing to sensory axon targeting in the olfactory bulb, supporting a model in which the coordinated expression of specific ORs and guidance related genes determine where sensory axons terminate in the bulb.

## Introduction

Despite tremendous progress in the identification of axonal guidance cues over the past few decades [1,2], there are only a limited number of developing neural systems in which axonal connectivity can be explained by the distributions of known cues [3–8]. Chief among these are sensory systems, as their orderly mapping of projections from source to target make them amenable to both recognizing relevant patterns of cue expression and detecting perturbations in axonal trajectories after cue alteration. Even among the best understood of these systems, there are significant gaps in our understanding of how cues guide axons to their targets. One difficulty is that some guidance cues remain to be identified, but perhaps equally important, so many different cues act simultaneously that their individual contributions are difficult to parse. Circumventing this problem is best accomplished in relatively simpler systems that have accessible embryos amenable to genetic manipulation.

These considerations motivated us to make a detailed study of sensory axon extension and targeting in the olfactory bulb of the developing zebrafish. Zebrafish have many advantages as a model system [9], but the olfactory system has the distinctive feature that an olfactory sensory neuron’s (OSN) identity is effectively reported by the singular odorant receptor (OR) it expresses. OSNs reside in the olfactory epithelia, which at early stages of development are comprised of bilateral pits. Each OSN chooses to express a single OR from a large gene repertoire [10–12], and the OR that is chosen is correlated with the target location of that OSN’s axon in the olfactory bulb [13–17]. OSN axons in zebrafish first project from the olfactory epithelium to specific, individually identifiable neuropils in the bulb called protoglomeruli [18]. OR-specific glomeruli later segregate from one another [19]. Zebrafish main olfactory bulb type ORs can be divided into three clades: A, B, and C, based upon their sequence similarity [13,20]. OSNs expressing clade A or B ORs project axons to the Central Zone (CZ) protoglomerulus, while OSNs expressing clade C ORs project to the Dorsal Zone (DZ) protoglomerulus [13]. An inference from these findings is that OSNs choosing to express clade A or B odorant receptors must also express different axonal guidance receptors than OSNs choosing clade C receptors.

In this study we identified and functionally tested potential guidance-related genes that are differentially expressed between groups of OSNs targeting different identifiable protoglomular neuropils in the olfactory bulb. We performed single-cell RNA sequencing (scRNA-seq) experiments from transgenically labeled OSNs collected from the developmental period when these neurons extend axons into the olfactory bulb [21–23]. Each OSN was categorized by the OR clade of its most predominantly expressed odorant receptor, and from this its expected protoglomerular target could be inferred. We then searched for differential gene expression patterns between OSNs with different targets. We identified differentially expressed transcription factors and candidate guidance-related genes. We then tested selected potential guidance related genes for guidance function. We identified four members of the delta family of non-clustered protocadherins as serving a role in olfactory sensory targeting in the olfactory bulb. Two members of the delta2-protocadherin family of adhesion molecules, *pcdh10b* and *pcdh17*, were found to be more highly expressed in OR clade A expressing and CZ protoglomerulus targeting OSNs; while two different members of the delta1-protocadherin family, *pcdh7b* and *pcdh11*, were found to be more highly expressed in OR clade C and DZ protoglomerulus targeting OSNs. We knocked down each one of these four protocadherin genes and examined the effect on OSN axon trajectories to and within the olfactory bulb. Loss of either *pcdh10b* or *pcdh17* induced axon targeting errors in OR clade A, CZ projecting OSNs; but did not induce targeting errors in OR clade C, DZ projecting OSNs. Conversely, loss of *pcdh7b* or *pcdh11* induced axon targeting errors in OR clade C, DZ projecting OSNs; but did not induce targeting errors in OR clade A, CZ projecting OSNs. The errors observed in these experiments are consistent with selective protocadherin-mediated homophilic adhesion. Interestingly, the delta1- and delta2-protocadherins have subtly different roles. The delta2-protocadherin *pcdh17* is required for normal axonal targeting to the correct location in the bulb, while the delta1-protocadherins *pcdh7b* and *pcdh11* help sensory axons infiltrate and incorporate into their correct target location in the bulb.

## Results

### Olfactory sensory neurons from three developmental timepoints contain similar cells that differ by maturity

**Fig 1:**
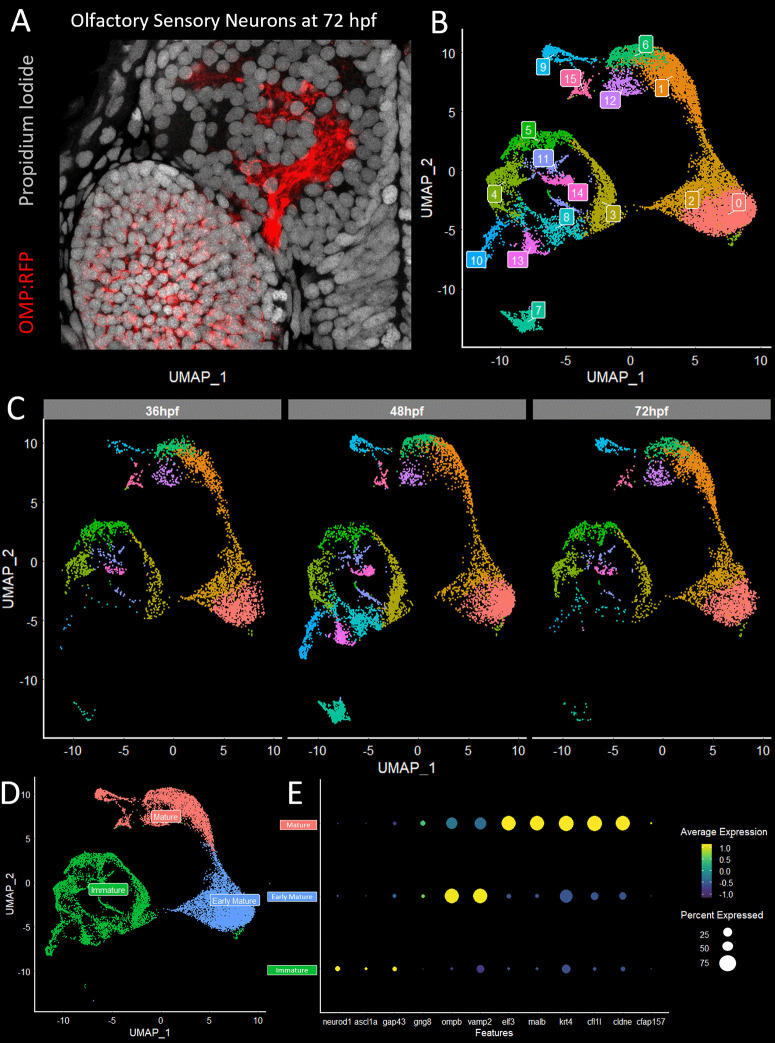
Single cell RNAseq analysis of olfactory sensory neurons in larval zebrafish. Individual OSNs from 36, 48, or 60 hours post fertilization (hpf) were isolated by FACS from transgenic fish olfactory epithelia expressing OMP promotor driven RFP and analyzed on the 10x Genomics platform. (A) OMP:RFP fluorescently labels a subset of olfactory sensory neurons that project axons to either the CZ, DZ, LG3 or MG protoglomeruli. Nuclei are stained with propidium iodide (Grey) and OSNs are labeled with OMP:RFP (red). (B) Data from OSNs isolated from 36, 48, and 72 hpf zebrafish were combined *in silico* and clusters were identified and visualized using Uniform Manifold Approximation and Projection (UMAP). (C) Visualization of UMAP clusters for each individual time point split out from the combined UMAP shows similar patterns of clustering for all three timepoints. (D) UMAP visualization of OSNs categorized and color-coded by their relative maturity using the markers described in E. Presumptive immature (green), early mature (blue), and mature (pink) OSNs are grouped together. (E) Marker genes that label OSNs at different stages of maturity were selected and visualized with a dot plot. Gene expression levels are represented on a viridis color scale with low expression (purple) and high expression (yellow). The percentage of cells expressing a particular gene is represented by circle circumference.

Olfactory epithelia were dissected from larval transgenic fish (Tg(-2.0ompb:LY-mRFP)^rw035a^ at 36, 48 and 72 hpf. In this line, hereafter referred to as OMP:RFP, the highly selective ciliated-type OSN marker olfactory marker protein (OMP) drives the expression of RFP [24] (Fig 1A). RFP expressing cells were separated from other cell types by FACS. Captured cells were processed through the 10x Genomics pipeline and the resulting reads were aligned to the GRCz11 version of the zebrafish genome [25]. A total of 21,718 cells were obtained of which 16,143 passed the following quality control parameters: [1] 800–7000 genes detected, [2] less than 50,000 reads per cell, and [3] less than 10% of reads belonging to mitochondrial genes. These criteria yielded 3,803 cells from 36hpf, 7,477 cells from 48hpf, and 4,863 cells from 72hpf samples.

Initial characterization was performed on each timepoint individually and included PCA analysis and clustering. OSNs obtained from all three timepoints contained similar clustered subsets of cells. When OSNs from each timepoint were combined and subjected to PCA analysis and clustering,16 unique clusters were identified and visualized using Uniform Manifold Approximation and Projection (UMAP) (Fig 1B). When this UMAP was re-populated by single sample timepoints, every cluster contained OSNs from each timepoint (Fig 1C).

Olfactory sensory neurons are continuously born throughout development and are continuously replenished in adulthood [26,27]. To explore whether cell maturity is a major determinant of clustering, the expression of previously identified marker genes for maturity was examined (Figs 1D,1E, and S1). Immature OSNs were identified by the expression of neuronal differentiation marker *neurod1* and the transcription factor *ascl1a* [28,29]. Immature OSNs transitioning to early mature neurons express the axonal outgrowth marker *gap43* [30]. Early mature OSNs are characterized by the emerging expression of the GPCR signaling pathway component *gng8,* high expression of *ompb,* and the presynaptic gene *vamp2* [31,32]. The most mature OSNs are characterized by high expression levels for several genes including *malb*, *krt4* (Keritin4), *cfl1l*, and *cldne* (Claudin E). Clusters 9 and 15 expressed cilia related genes including cfap157. The early mature and mature groupings resemble recent data from [33]. The overall distribution of markers and expression trajectory analysis supports the classification of OSNs by a developmental sequence from immature, early mature, to mature groupings.

DESeq2 was used to identify genes differentially expressed between clusters. One cluster, cluster 7, contained immune cells as assessed by the expression of the T-cell markers *tnfb*, *cxcr3.3,* and *CD83* (S2 Fig) [34–36]. We speculate that these are phagocytic immune cells that are indirectly labeled by ingesting RFP from dying OSNs. These cells were removed from subsequent analysis. The expression of different individual main olfactory bulb type ORs, OR subfamilies, and OR homology clades were all indiscriminately distributed between clusters.

### Odorant receptors co-expressed in the same OSN generally belong to the same OR subfamily

**Fig 2:**
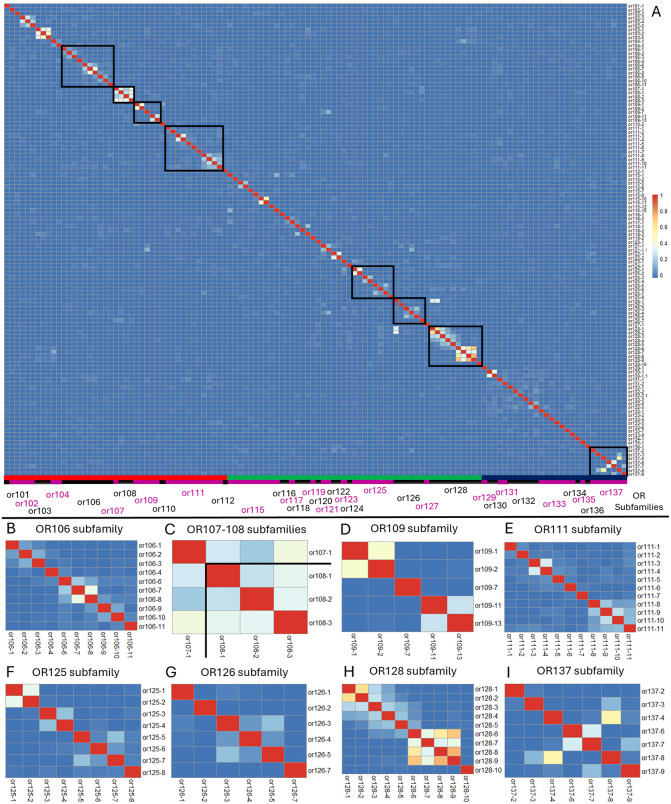
Co-expression of odorant receptors in olfactory sensory neurons. Nonparametric Spearman correlation coefficients were calculated for every pair of ORs in OSNs expressing more than one odorant receptor. (A) Positive correlations are visualized as warmer colors. Each row and column represent an individual odorant receptor (labeled below and on right side). Below the heatmap, alternating black and magenta bands indicate groups of ORs from the same OR subfamily. The red, green, and blue line just above indicates OR clades A, B, or C respectively. Black boxes in (A) surround OR subfamilies that are shown in greater detail below. (B-I).

Mature olfactory sensory neurons are thought to express a single odorant receptor from a large repertoire of OR genes in a monoallelic manner [10,12,37–39]. Multiple studies have shown that OSNs can transiently express more than one OR during development [40–43]. We characterized the co-expression of ORs in individual OSNs in larval zebrafish. Of the 16,161 OMP:RFP positive cells in this study, no OR transcript was detected in 4,299 cells, 4,314 cells expressed transcripts from a single OR gene, and 7,548 cells expressed transcripts from two or more OR genes. ORs are organized into clustered gene subfamilies that reside in a limited number of chromosomal locations within the genome [44]. We constructed a co-expression matrix to determine which ORs were co-expressed with one another. We found that co-expression occurs most frequently between adjacent genes within the same OR subfamily (Fig 2A).

The OR co-expression matrix reveals that almost all significant co-expression occurs within subfamily gene clusters (some examples are boxed (Fig 2A boxes and 2B-2I). Within a subfamily, some blocks of genes are more often co-expressed than others. Some examples include or106-6 to or106–8 (Fig 2B), or108-1 to or108-3 (Fig 2C), or109-1 and or109-2 (Fig 2D), or111–8 to or111–11 (Fig 2E), or125-1 and or125-2 (Fig 2F), or126-3 to or126-5 (Fig 2G), and or128-6 to or128–9 (Fig 2H). These blocks are composed of genes that are immediately adjacent to one another in the genome, suggesting that their proximity exposes them to some of the same genetic control elements that initiate gene transcription. There were two notable instances in which ORs from different subfamilies are co-expressed. Or126-1 was sometimes co-expressed with either or128-1 or or128-2. Although these OR genes are assigned to different subfamilies based on their sequences, they are immediately adjacent to each other on chromosome 15 and may be influenced by similar transcriptional control regions. The second instance of co-expression between members of different subfamilies is harder to explain. ORs 107-1, 108-1, 108-2, and 108-3 are relatively frequently co-expressed with one another. Or107-1 is currently mapped to chromosome 15 while the or108 subfamily gene cluster is mapped to chromosome 10. We considered the possibility that this apparent co-expression is the result of mistaken assignments of OR identity based on similarities between related receptors. This is very unlikely as the C-termini of or107 and or108 sequences are not very similar. Any sequences that matched more than one gene target were automatically rejected in our overall analysis and ambiguous gene assignments should have been very rare.

These observations suggest that OR gene co-expression is nearly always confined to genes within an OR subfamily before feedback mechanisms further restrict expression to a singular OR allele [10,45–49]. This might occur as nearby cis enhancer elements, or an active hub of multiple trans enhancer elements, initiate OR expression [50–52]. It has recently been reported that more than one OR enhancer hub can form within the nucleus of an OSN [51]. Perhaps only one of these is active enough to drive substantial OR expression as the coactivated OR loci we detected are nearly always immediately adjacent to each other. It should also be noted that co-expression of ORs from different clades is very rare, so that the categorization of OSNs by the most highly expressed OR that we describe below, should be unaffected by the pattern of co-expression we observe.

### Differential expression of genes in OSNs expressing odorant receptors from different OR clades

Previous work has shown that the initial protoglomerular target of an OSN axon is related to the clade of their chosen OR [13]. OSNs expressing ORs from clades A or B project to the CZ protoglomerulus, while OSNs expressing ORs from clade C project to the DZ protoglomerulus. This suggests coordination between OR choice and the expression of axon guidance-related molecules. We grouped OSNs based upon the OR they predominantly expressed. Identity was assigned if an OSN expressed only one OR, or if the most highly expressed OR was expressed at a level 5 or more times that of the second most highly expressed OR. OSNs that did not meet these criteria were eliminated from further analysis. A total of 7,308 cells were assigned OR identities. OSNs expressing ORs from the same clade were then grouped together for differential expression analysis. OSNs from the less mature group contributed 47% of the cells that were included, while the immature and most mature groups contributed 26% and 27% respectively. The analysis described below gave similar results regardless of whether the cells from the immature group were included.

**Fig 3:**
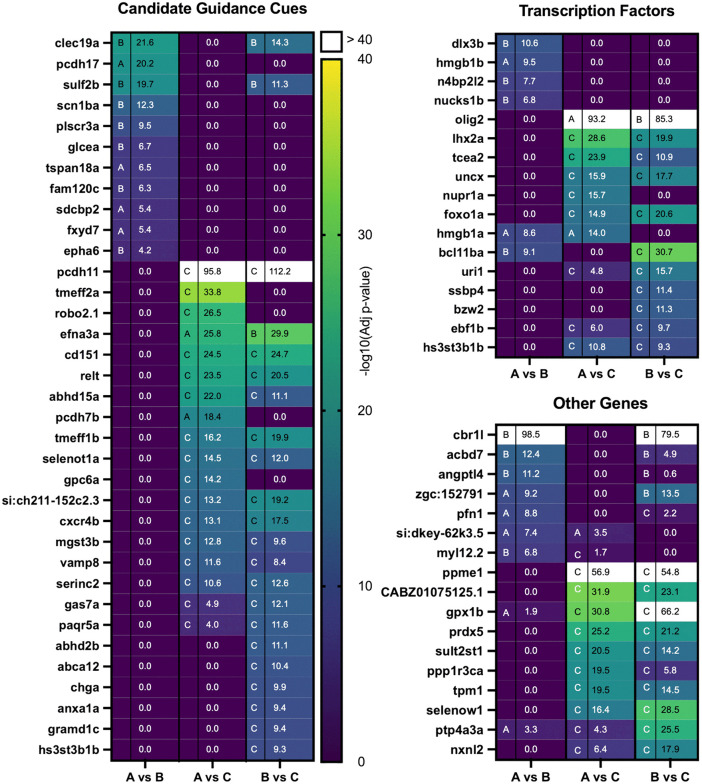
Differentially expressed genes in olfactory sensory neurons that express ORs from different homology clades. OSNs were grouped by the homology clade (A, B, or C) of their predominantly expressed OR. DESeq2 was then used to identify differentially expressed genes between the three groups. Each column represents a separate clade comparison with clades A vs B on the left, clades A vs C in the middle, and clades B vs C on the right. The degree of differential expression is ranked according to the -log10 adjusted p-value for each comparison and represented on a virdis color scale with the highly-differentially expressed genes (yellow) and non-differentially expressed genes (purple). To improve visual clarity, the most highly-differentially expressed genes are labeled white if their -log10 adjusted p-value > 40. The clade in which the indicated gene is more highly expressed is indicated by a single letter corresponding the clade. **(Left)** Candidate guidance cues include genes containing signal sequences or transmembrane domains. **(Top Right)** Transcription factors were identified as genes with known or predicted DNA binding activity. **(Bottom Right)** Other differentially expressed genes including enzymes, ion channels, and cytoskeletal genes.

OSNs were sorted into three groups corresponding to the homology clade of their predominant OR. DESeq2 was used to identify genes differentially expressed between these three groups. Each group was tested against the others, generating three comparisons: OSNs expressing OR clades A vs B, A vs C, and B vs C. These comparisons identified genes differentially expressed between each pair of clades. To enrich for potential guidance cues and their receptors, genes were manually curated to include those that were both differentially expressed and contained either a signal sequence or a transmembrane domain (Fig 3, Left column). This curated list of genes includes known guidance cues, genes with known roles in olfactory development, and potential novel guidance cues and/or receptors. Ephs and Ephrins differentially expressed in clades A and B have previously been shown to play a role in the development of the mouse olfactory system. EphA6:Ephrin-A5 signaling helps segregate anterior and posterior projecting vomeronasal OSNs in the mouse [53]. Loss of either *ephrin-A5* or *ephrin-A3* induces posterior shifts in glomerular innervation of p2 or SR1 OR expressing OSNs in the mouse main olfactory bulb [54]. Conversely, their overexpression induces an anterior shift in targeting. In mice, Slit/Robo2 repulsion helps to organize glomerular position along the dorsal-ventral axis of the olfactory bulb [55,56]. *Robo2* is more highly expressed in zebrafish OR clade C as compared to OR clade A OSNs. Both the loss of Robo2, or the general overexpression of its ligand Slit2, have been shown to cause dramatic ectopic midline crossings of OSN axons in the developing zebrafish olfactory system [57]. OR clade C BAC:OR130–1 OSN axons are more likely to terminate ectopically in *robo2* mutant fish [58]. Morpholino knockdown experiments in zebrafish have shown that *cxcr4b*, most highly expressed in clade C OR expressing OSNs, is required for normal olfactory placode development and successful OSN axon projection from the olfactory epithelium to the olfactory bulb [59].

A previous fluidigm C1 based scRNA-seq experiment identified similar differentially expressed axonal guidance receptors important for protoglomerular targeting [58]. This older study sampled a small number of OSNs but probed gene expression at a far greater read depth. The current study confirms the previous finding that *nrp1a* is most highly expressed in OR clade A expressing OSNs, (p = 3.49E-05 Clade A > C) while *robo2* is most highly expressed in OR clade C expressing OSNs (p = 3.38E-27 Clade C > A). Additionally, both studies identified *sulf2b* as most highly expressed in OR clade B expressing OSNs, while *anax1a* is most highly expressed in OR clade C expressing OSNs. Thus, the candidate genes identified here largely confirm our previous findings while expanding the list of potential guidance-related genes.

### Differential expression of delta-protocadherin family members

This study focuses on one family of adhesion molecules whose members are differentially expressed between OSNs, the non-clustered delta-protocadherins. Our earlier scRNA-seq experiment identified *pcdh11* as most highly expressed in OR clade C expressing OSNs and the current study confirms this finding. In this study we also found *pcdh7b* to be more highly enriched in OR clade C as compared to OR clade A OSNs. In contrast, *pcdh10b* is most highly enriched in OR clade A as compared to OR clade C expressing OSNs. Similarly, *pcdh17* is more highly enriched in OR clade A as compared to OR clade B expressing OSNs. As described below, we tested these four genes for guidance activity in the developing olfactory system.

Other genes including transcription factors were differentially expressed between OSNs expressing odorant receptors from different OR clades. Transcription factor *olig2* was highly expressed in OR clades A and B as compared to OR clade C expressing OSNs. Recently *olig2* has been shown to regulate the expression of semaphorins, which have known axon guidance effects in the zebrafish olfactory system [60]. Transcription factor *lhx2a* was most highly expressed in OR clade C expressing OSNs. OSNs in *lhx2* null mice fail to fully differentiate in regionally restricted areas of the olfactory epithelium and *lhx2a* has been shown to act as a master regulator of multiple OR genes [50,51,61,62]. Whether it might differentially affect the expression of ORs from specific clades in fish is unknown.

### *Pcdh11* is required for normal DZ protoglomerular targeting

As *pcdh11* is most highly expressed in OSNs choosing homology clade C ORs (Fig 3), we tested the necessity of *pcdh11* for axon targeting in these OSNs. *Pcdh11* was knocked down using a CRISPR-based approach. Just-fertilized zebrafish embryos injected with ribonucleoprotein (RNP) complexes composed of Cas9 and three different guide RNAs (gRNAs) that target different parts of the same gene can effectively knockdown the targeted gene [63,64]. If RNPs are designed and selected for their ability to introduce CRISPR induced frame-shifting indels, the resulting F0 knockdown embryos have been found to phenocopy null mutant embryos. We used this approach to compare the axon trajectories of selectively labeled OSNs in uninjected control embryos to F0 *pcdh11* knockdown embryos. The axons targeting specific protoglomeruli were visualized using two BAC transgenic lines that label distinct subsets of OSNs which have chosen to express odorant receptors or111–7 or or130–1. A line containing Tg(BAC:OR111–7:Gal4) (henceforth referred to as BAC:OR117) crossed with a line containing the fluorescent reporter Tg(UAS:gap43-Citrine) (referred to as UAS:Citrine) label a small number of OR clade A expressing OSNs whose axons project to the ventral and medial CZ protoglomerulous. A line containing Tg(BAC:OR130–1:Gal4) (referred to as BAC:OR130–1) crossed with UAS:Citrine label a small number of OR clade C expressing OSNs whose axons project to the more dorsal and lateral DZ protoglomerulous. These marker lines enable us to characterize the accuracy of OSN axon trajectories in both uninjected control and F0 knockdown embryos (S3 Fig).

**Fig 4:**
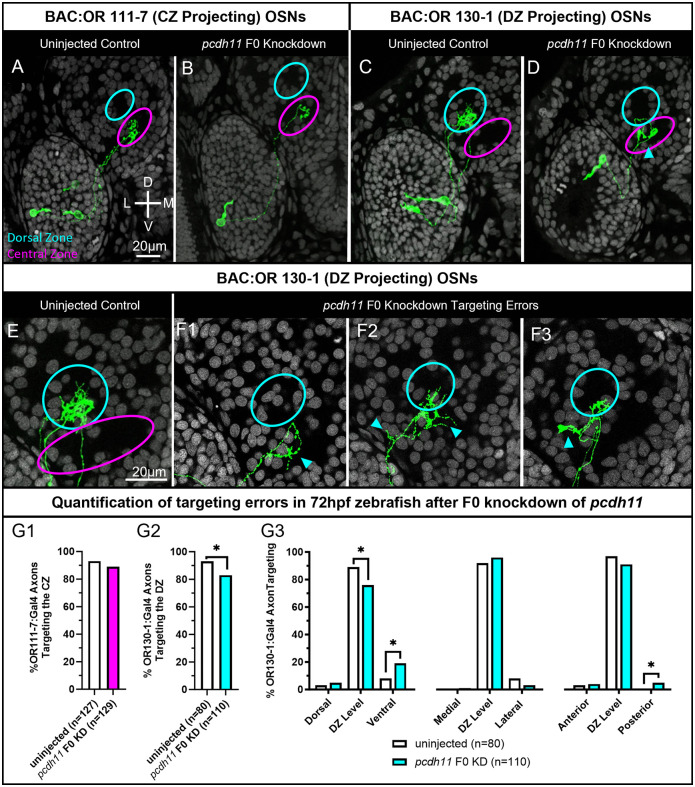
F0 Knockdown of *pcdh11* disrupts axonal projections to the DZ protoglomerulus. OSN axon trajectories of uninjected embryos and sibling embryos injected with 3 CRISPRs that knockdown *pcdh11* were compared at 72hpf. (A,B) The axons of BAC:OR111–7 labeled OSNs (green) terminate in the CZ protoglomerulus (magenta) in both uninjected and *pcdh11* Fo knockdown conditions. (C,D) The axons of BAC:OR130–1 labeled OSNs terminate in the DZ protoglomerulus (cyan) in uninjected embryos but sometimes terminate more ventrally in the CZ protoglomerulus in F0 knockdown conditions. (E,F) Higher magnification images of BAC:130–1 labeled axon trajectories within the olfactory bulb. (E) Uninjected sibling control. (F1,F2,F3) Examples of BAC:130–1 labeled axons terminating in ectopic locations in *pcdh11* knockdowns. Cyan arrows highlight errors. (G1) Percentages of BAC:OR111–7 labeled axons correctly terminating in the CZ protoglomerulus in control and knockdown conditions. (G2) Percentages of BAC:OR130–1 axons correctly terminating within the DZ protoglomerulus in control and knockdown conditions. (G3) BAC:OR130–1 labeled axons in knockout embryos were more likely to terminate in ectopic ventral or posterior locations in relation to the DZ protoglomerulus than in uninjected controls. Statistical comparisons were performed using a one-tailed Fisher’s Exact test with * denoting p ≤ 0.05.

CRISPR RNPs for *pcdh11* were selected by their predicted efficiency and mutagenic ability [65]. Their efficacy was directly tested by sequencing segments of the *pcdh11* gene targeted by CRISPRs and subjecting the sequence data to analysis by DECODR [66]. Assuming they act independently, the three RNPs targeting *pcdh11* should together introduce alterations with frame shifts or premature stops in 99% of targeted loci in injected embryos (Table 1). F0 knockdown of pcdh11 does not affect the axon targeting of OSNs expressing BAC:OR111–7. Axons project to the CZ protoglomerulus with few errors in both sibling uninjected and in *pcdh11* F0 knockdown embryos (Fig 4A, 4B, and 4G1, magenta). The trajectories of *pcdh11* F0 knockdown and uninjected controls are the same for all parameters. In contrast, F0 knockdown of pcdh11 induces a higher rate of targeting errors in BAC:OR130–1 labeled axons as compared to uninjected sibling controls (Fig 4C and 4D). The percentage of BAC:OR130–1 axons successfully targeting the DZ protoglomerulus is significantly reduced as compared to uninjected controls (Fig 4G2, cyan). The accuracy of BAC:OR130–1 axon projections was further characterized by the locations of their terminations relative to the DZ protoglomerulus (Fig 4G3). BAC:OR130–1 axons in *pcdh11* F0 knockdowns have significantly elevated levels of ectopic ventral and posterior terminations relative to the dorsal zone protoglomerulus (Fig 4F1, 4F2, 4F3, cyan arrows, and 4G3). Processes were observed within the DZ that turned away and ultimately terminated more ventrally. These errors create a looping trajectory as axons extended into the DZ and then loop back to terminate more ventrally (Fig 4F1 and 4F3).

**Table 1:** Effectiveness of CRISPR F0 knockdowns determined by DECODR. For each target protocadherin the effectiveness of the top three CRISPR RNPs was assessed by DECODR on pools of 10 embryos at 48hpf. The knockout score represents the frequency at which a frameshift mutation or a premature stop was introduced.

	crRNA Sequence	Pam Seq.	crRNA location (GRCz11)	% Non-Frameshift	% Frameshift	Knockout Score
***pcdh7b***						
Target 1	TACATTACCTACCCGGACAT	CGG	chr7–6332593763325956	57	43	43
Target 2	CGGCTGCTTCTCGCCGTCTG	TGG	chr7–6332648663326505	20	80	80
Target 3	GAGGGAGGGCACCTAGTCTG	GGG	chr7–6345820363458222	28	72	80
*pcdh7b* Total Estimated Knockout Score = 98%						
***pcdh10b***						
Target 1	GTGCCCGAGGAAGCGGAGCA	TGG	chr14–4300035343000372	7	93	93
Target 2	GGGAGGCTGGCCACCGTCCA	CGG	chr14 + 4299981242999831	18	82	83
Target 3	TGGCGTTGGACCACAGTTCG	GGG	chr14 + 4299882742998846	60	40	40
*pcdh10b* Total Estimated Knockout Score = 99%						
***pcdh11***						
Target 1	TCAAGTTTAGGTCTTTGCGC	AGG	chr14 + 2728885927288878	31	69	69
Target 2	GGGTCAAATGCGGAAGGGAC	TGG	chr14 + 2728851027288529	36	64	64
Target 3	TCGAGTGGTCTCATAATCTA	AGG	chr14 + 2728244227282461	15	85	95
*pcdh11* Total Estimated Knockout Score = 99%						
***pcdh17***						
Target 1	TCCGACCTTGGAGAGCTGAG	GGG	chr11 + 3272001132720030	3	97	100
Target 2	CTGGCCCATGACCCAGATTT	AGG	chr11–3272093532720954	43	57	61
Target 3	GCAATGGGCAGCTCCAGTGT	AGG	chr11–3272122532721244	9	91	91
*pcdh17* Total Estimated Knockout Score = 100%						

We compared the F0 knockdown phenotype to *pcdh11* mutant embryos obtained from the Zebrafish International Resource Center (ZIRC). This mutant (sa31987) was generated by ENU mutagenesis and has a point mutation that generates a premature stop in exon 1. *Pcdh11* mutant fish were crossed to each of the transgenic lines BAC:OR111–7:Gal4, BAC:OR130–1:Gal4, or UAS:Citrine and the progeny were maintained as heterozygotes. Fish containing either *pcdh11* + /-; BAC:OR111–7:Gal4 or *pcdh11* + /-;BAC:OR130–1:Gal4 were crossed to fish containing *pcdh11* + /-;UAS:Citrine. Citrine labeled axon trajectories and protoglomerular targeting in *pcdh11* + /+ and -/- progeny were compared.

**Fig 5:**
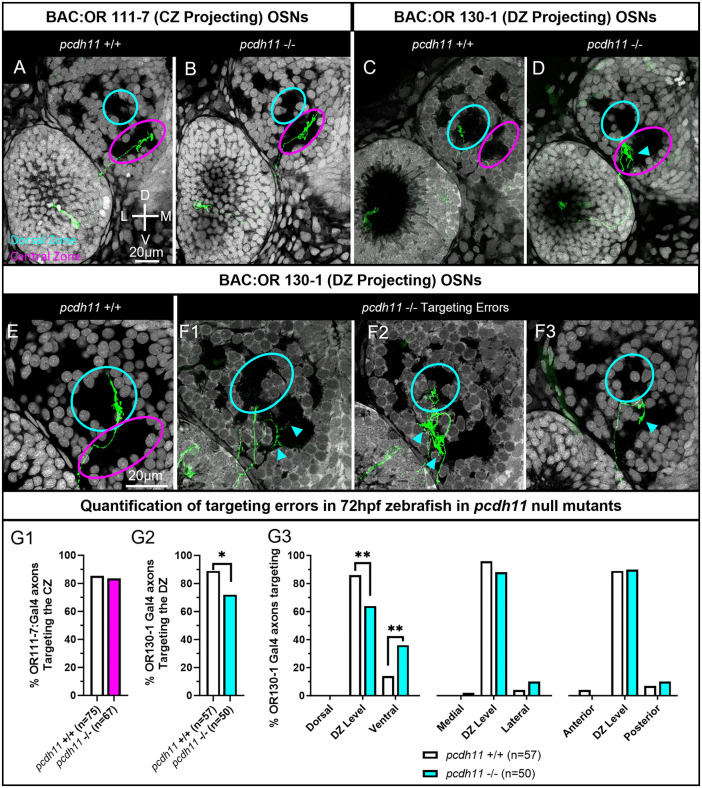
*Pcdh11* null mutants replicate the *pcdh11* F0 knockdown phenotype. OSN axon trajectories in Wild Type (WT) *pcdh11*+/+ and sibling *pcdh11 -/- mutants* were compared at 72hpf. (A,B) The axons of BAC:OR111-7 labeled OSNs (green) terminate in the CZ protoglomerulus (magenta) in both WT and *pcdh11* mutant embryos. (C,D) The axons of BAC:OR130-1 labeled OSNs terminate in the DZ protoglomerulus (cyan) in WT embryos but sometimes terminate more ventrally in the CZ protoglomerulus in *pcdh11* mutant embryos(cyan arrow). (E,F) Higher magnification images of BAC:130-1 labeled axon trajectories within the olfactory bulb. (E) Wild type control. (F1,F2,F3) Examples of BAC:130-1 labeled axons terminating in ectopic locations in *pdch11* -/- embryos. Cyan arrows highlight errors. (G1) Percentages of OR111-7 labeled axons correctly terminating in the CZ protoglomerulus in WT and *pcdh11* mutant embryos. (G2) Percentages of OR130-1 axons correctly terminating in the DZ protoglomerulus in WT and *pcdh11* mutant embryos. (G3) BAC:OR130-1 labeled axons in knockout embryos were more likely to terminate in ectopic ventral locations in relation to the DZ protoglomerulus than in wild type controls. Statistical comparisons were performed using a one tailed Fisher’s Exact test with * denoting p ≤ 0.05 and ** p ≤ 0.01.

BAC:OR111–7 expressing OSNs correctly project axons the CZ protoglomerulus in both *pcdh11* mutants and sibling wild type controls (Fig 5A, 5B, and 5G1). In contrast, the percentage of BAC:OR130–1 axons terminating in the DZ protoglomerulus is significantly reduced in *pcdh11* mutants as compared to sibling controls (Fig 5C, 5D, and 5G2). BAC:OR130–1 axons aberrantly project to regions ventral to their DZ protoglomerular target, frequently terminating in or near the CZ protoglomerulus (Fig 5F1-5F3, cyan arrows, and 5G3).

BAC:OR130–1 axon targeting errors in both *pcdh11* mutants and *pcdh11* F0 knockdowns are very similar. In both data sets BAC:OR130–1 axons first project to the general vicinity of their normal protoglomerular target but terminate in unusual ventral locations (Figs 4G3 and 5G3). Misprojecting axons often show similar trajectories characterized by axons approaching or entering their dorsal zone target before turning away and terminating ventrally (Figs 4D, 4F1, 4F3, 5D, and 5F3). The consistency between *pcdh11* F0 knockdown and mutant phenotypes validates using F0 knockdowns to assess loss of function phenotypes for axon guidance-related genes in this system. These results suggest that BAC:OR130–1 expressing OSNs require *pcdh11* to enter and remain within the dorsal zone protoglomerulus.

### *Pcdh7b* is required for normal DZ protoglomerular targeting

**Fig 6:**
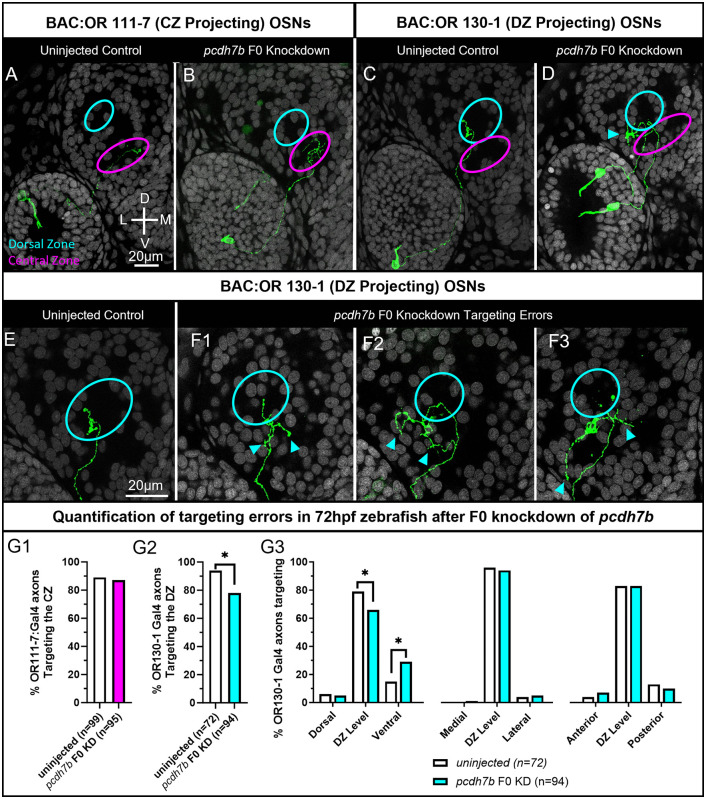
F0 knockdown of *pcdh7b* disrupts axonal projections to the DZ protoglomerulus. OSN axon trajectories of uninjected embryos and sibling embryos injected with 3 CRISPRs that knockdown *pcdh7b* were compared at 72hpf. (A,B) The axons of BAC:OR111-7 labeled OSNs (green) terminate in the CZ protoglomerulus (magenta) in both uninjected and *pcdh7b* F0 knockdown conditions. (C,D) The axons of BAC:OR130-1 labeled OSNs terminate in the DZ protoglomerulus (cyan) in uninjected embryos but sometimes terminate more ventrally in the CZ protoglomerulus in F0 knockdown conditions. (E,F) Higher magnification images of BAC:130-1 labeled axon trajectories within the olfactory bulb. (E) Wildtype control. (F1,F2,F3) Examples of BAC:OR130-1 labeled axons terminating in ectopic locations in knockdown embryos. Cyan arrows highlight errors. (G1) Percentages of OR111-7 labeled axons correctly terminating in the CZ protoglomerulus in control and knockdown conditions. (G2) Percentages of BAC:OR130-1 axons correctly terminating within the DZ protoglomerulus in control and knockdown conditions. (G3) BAC:OR130-1 labeled axons in knockout embryos were more likely to terminate in ectopic ventral locations in relation to the DZ protoglomerulus than in uninjected controls. Statistical comparisons were performed using a one tailed Fisher’s Exact test with * denoting p ≤ 0.05.

Like *pcdh11*, *pcdh7b* is more highly expressed in OR clade C expressing OSNs (Fig 3). To test if *pcdh7b* is required for the correct targeting of OSN axons, *pcdh7b* F0 knockdown experiments were performed in both BAC:OR111–7 (Clade A) and BAC:OR130–1 (Clade C) labeled OSNs. Assuming they act independently, the three CRISPR RNPs targeting *pcdh7b* should together introduce indels with frame shifts or premature stops in 97% of targeted loci in injected embryos (Table 1). F0 knockdown of *pcdh7b* does not affect the ability of BAC:OR111–7 OSNs to correctly target the CZ protoglomerulus. BAC:OR111–7 labeled axons correctly target the CZ protoglomerulus at similar rates in both uninjected controls and *pcdh7b* F0 knockdown embryos (Fig 6A, 6B, and 6G1). In contrast, BAC:OR130–1 labeled axons target the DZ protoglomerulus at normal rates in uninjected embryos but terminate in the DZ protoglomerulus at a significantly reduced rate in *pcdh7b* F0 knockdown embryos (Fig 6C, 6D, and 6G2). Mistargeted BAC:OR130–1 labeled axons have numerous terminations outside the DZ (6F1, 6F2 and 6F3 and 6F3, cyan arrows). BAC:OR130–1 labeled axons have a significant increase in the number of ectopic ventral axon terminations in *pcdh7b* F0 knockdown embryos as compared to uninjected controls (Fig 6G3).

### Simultaneous knockdown of *pcdh11* and *pcdh7b.*

**Fig 7:**
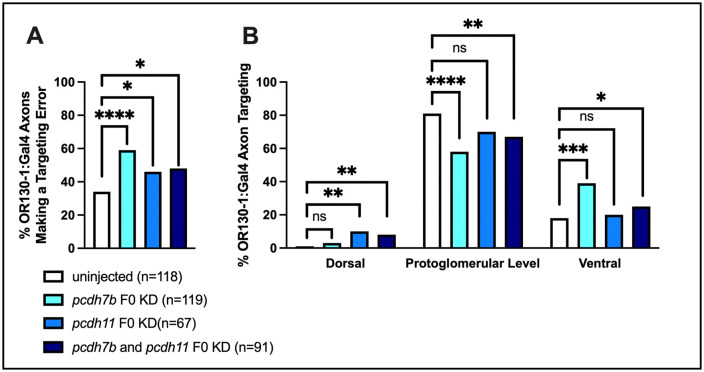
Errors induced by the knockdown of *pcdh7b* and *pcdh11* are not additive. BAC:OR130-1 labeled sensory axon trajectories in uninjected, *pcdh7b* knockdown, *pcdh11* knockdown, or *pcdh7b*/*pcdh11* double knockdown sibling embryos were compared at 72hpf. (A) Percentages of OSNs with axons making at least one error are elevated to the same degree in all knockout conditions as compared to uninjected controls. (B) Targeting errors in the knockdown conditions were predominantly directed to ectopic ventral locations in relation to the DZ protoglomerulus. Statistical comparisons were performed using a one tailed Fisher’s Exact test with * denoting p ≤ 0.05, ** denoting p ≤ 0.01, *** denoting p ≤ 0.001, and **** denoting p ≤ 0.0001.

A comparison of BAC:OR130–1 axon trajectories in *pcdh7b*, *pcdh11, and pcdh7b/pcdh11 double knockdowns* was performed to determine if their combined loss would produce additive or synergistic effects. To avoid the toxicity that can occur when too many different guide RNAs are injected into embryos, the two best CRISPR guides for *pcdh7b* (Target 2 and Target 3), the two best guides for *pcdh11* (Target 1 and Target 3), or all four of these guides were injected into fertilized embryos at the one cell stage. We sequenced targeted regions from a pool of 5 embryos to determine the knockdown efficacy from each injected batch used to score axonal trajectories. The sequencing results indicated that knockdowns were effective between 76% and 99% for *pcdh7b*, and between 86% and 95% for *pcdh11*. This is a lower rate than observed when three guides are used for each gene. As before, there is a statistically significant increase in mistargeting of BAC:OR130–1 labeled axons in *pcdh7b* or in *pcdh11* single knockdowns as compared to uninjected controls (Fig 7A). We also observe increased mistargeting of BAC:OR130–1 labeled axons in *pcdh7b*/*pcdh11* double knockdowns as compared to uninjected controls, but there is no additional mistargeting of BAC:OR130–1 labeled axons in *pcdh7b*/*pcdh11* double knockdowns as compared to either *pcdh7b* or *pcdh11* single knockdowns. The predominant errors in BAC:OR130–1 labeled axons in all knockdown conditions are processes escaping from the DZ protoglomerulus after entry, rather than a failure of axons to reach the protoglomerulus (Fig 7B). Escaping processes were predominantly directed ventrally away from the DZ protoglomerulus.

### *Pcdh17* is required for normal CZ protoglomerular targeting

**Fig 8:**
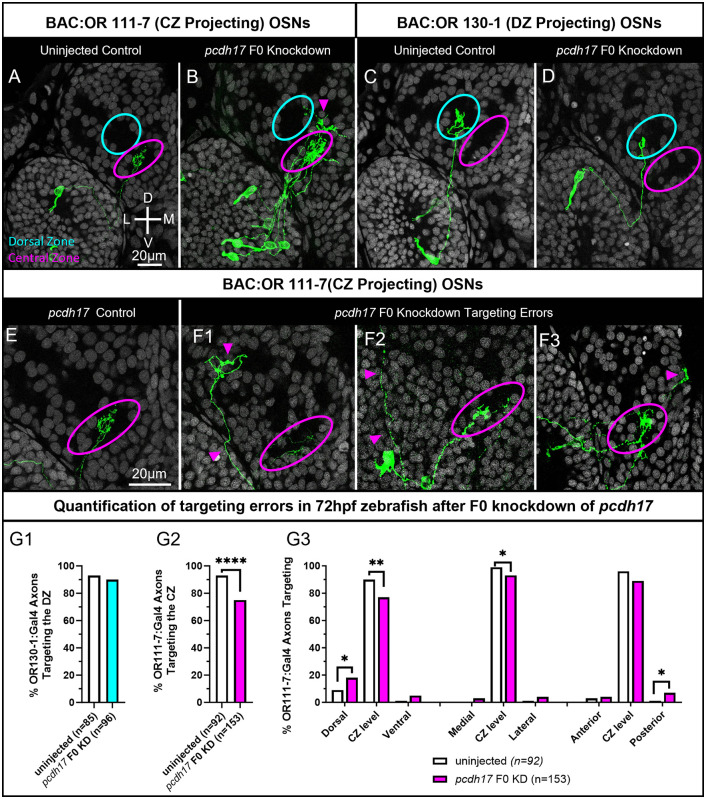
F0 knockdown of *pcdh17* disrupts axonal projections to the CZ protoglomerulus. OSN axon trajectories of uninjected embryos and sibling embryos injected with 3 CRISPRs that knockdown *pcdh17* were compared at 72hpf. (A,B) The axons of BAC:OR111-7 labeled OSNs (green) terminate in the CZ protoglomerulus in uninjected embryos (magenta). They sometimes terminate outside the CZ protoglomerulus in *pcdh17* F0 knockdown embryos. (C,D) The axons of BAC:OR130-1 labeled axons terminate in the DZ protoglomerulus (cyan) in uninjected and F0 knockdown embryos. (E,F) Higher magnification images of BAC:OR111-7 labeled axon trajectories within the olfactory bulb. (E) Uninjected control. (F1,F2,F3) Examples of BAC:OR111-7 labeled axons terminating in ectopic locations in knockdown embryos. Cyan arrows highlight errors. (G1) Percentages of BAC:OR130-1 labeled axons correctly terminating in the DZ protoglomerulus in control and knockdown conditions. (G2) Percentages of BAC:OR111-7 axons correctly terminating in the CZ protoglomerulus in control and knockdown conditions. (G3) BAC:OR111-7 labeled axons in knockout embryos were more likely to terminate in ectopic dorsal and posterior locations in relation to the CZ protoglomerulus than in uninjected controls. Statistical comparisons were performed using a one-tailed Fisher’s Exact test with * denoting p ≤ 0.05, ** p ≤ 0.01 and **** p ≤ 0.0001.

In contrast to *pcdh7b* and *pcdh11*, *pcdh17* is most highly expressed in OR clade A expressing OSNs that project to the CZ protoglomerulus (Fig 3). *Pcdh17* F0 knockdown experiments were performed in both OR clade A BAC:OR111–7 and clade C BAC:OR130–1 transgenic lines. Assuming they act independently, the three RNPs targeting *pcdh17* should together introduce indels with frame shifts or premature stops in 100% of targeted loci in injected embryos (Table 1). The ability of BAC:OR111–7 axons to correctly target the CZ protoglomerulus is significantly reduced in *pcdh17* knockdowns as compared to uninjected controls (Fig 8A, 8B, and 8G2). Mistargeting axons are found to extend in multiple ectopic directions (Fig 8B). In some embryos, BAC:OR111–7 axon misprojections fail to enter the olfactory bulb, project large distances, and terminate far from their normal CZ protoglomerular target (Fig 8F1 and 8F2, magenta arrows). Other misprojections within the bulb include ectopic dorsal and medial terminations in which axons reach but fail to remain within the CZ (Fig 8B and 8F3, magenta arrow). BAC:OR111–7 expressing OSNs show an increase in dorsal and posterior misprojections in *pcdh17* knockdowns as compared to controls (Fig 7G3). In contrast, BAC:OR130–1 axons exhibit no deficits in DZ protoglomerular targeting in *pcdh17* knockdowns when compared to uninjected controls (Fig 8C, 8D, and 8G1).

### F0 knockdown of *pcdh10b* disrupts axonal targeting

**Fig 9:**
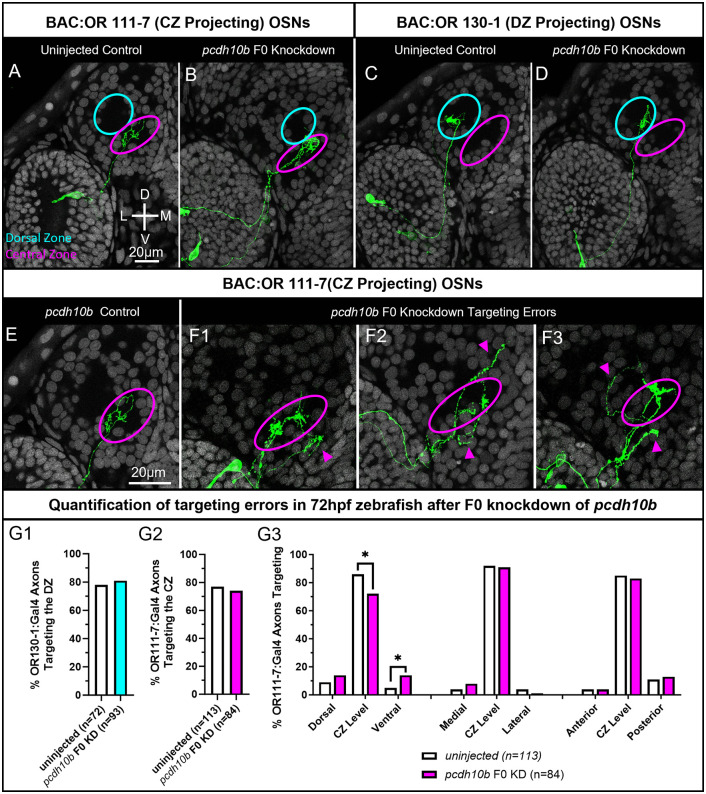
F0 knockdown of *pcdh10b* disrupts axonal projections to the CZ protoglomerulus. OSN axon trajectories of uninjected embryos and sibling embryos injected with 3 CRISPRs that knockdown *pcdh10b* were compared at 72hpf. (A,B) The axons of BAC:OR111-7 labeled OSNs (green) terminate in the CZ protoglomerulus in uninjected embryos (magenta). They sometimes have axonal processes that terminate outside the CZ protoglomerulus in *pcdh10b* F_o_ knockdown embryos. (C,D) The axons of BAC:OR130-1 labeled axons terminate in the DZ protoglomerulus (cyan) in uninjected and F0 knockdown embryos. (E,F) Higher magnification images of BAC:OR111-7 labeled axon trajectories within the olfactory bulb. (E) Uninjected control. (F1,F2,F3) Examples of BAC:OR111-7 labeled axons terminating in ectopic locations in knockdown embryos. Cyan arrows highlight errors. (G1) Percentages of BAC:OR130-1 labeled axons correctly terminating in the DZ protoglomerulus in control and knockdown conditions. (G2) Percentages of BAC:OR111-7 axons with terminations in the CZ protoglomerulus in control and knockdown conditions. (G3) BAC:OR111-7 labeled axons in knockout embryos were more likely to have ectopic terminal processes outside the CZ protoglomerulus than uninjected controls, even if they had terminations within the CZ (G2). Statistical comparisons were performed using a one tailed Fisher’s Exact test with * denoting p ≤ 0.05.

Of the four protocadherins we tested for OSN targeting defects in knockdowns, *pcdh10b* displayed the weakest level of differential expression between OSNs expressing ORs from different clades. DESeq2 revealed that *pcdh10b* may be slightly enriched in OR clade A OSNs as compared to clade C OSNs (adj p-value = 0.0047), and in OR clade B OSNs as compared to clade C OSNs (adj p-value = 0.014). Further comparisons in which OSNs from OR clades A and B were combined and compared to OSNs from OR homology clade C showed a stronger differential expression (adj p-value = 3.34 x10 -5). Assuming they act independently, the three RNPs we used to knockdown *pcdh10b* should together introduce indels with frame shifts or premature stops in 99% of targeted loci in injected embryos (Table 1). OR clade C BAC:OR130–1 labeled axons correctly target the DZ protoglomerulus in *pcdh10b* F0 knockdowns and uninjected controls (Fig 9C, 9D, and 9G1). OR clade A BAC:OR111–7 axons reach the CZ protoglomerulus in both *pcd10b* F0 knockdown embryos and uninjected controls, however, some of these axons have ectopic processes (Fig 9A, 9B, and 9F1-9F3 arrows). Errors include axons occasionally mistargeting ventrally (Fig 9F1 and 9F3 arrows), or axons that misproject dorsally (Fig 9F2 and 9F3 arrows). While the overall ability of BAC:OR111–7 axons to navigate to the CZ protoglomerulus was unaffected, some axons exhibited abnormal projections and failed to remain within the CZ.

### Delta-protocadherins are expressed in distinct patterns within the olfactory bulb

**Fig 10:**
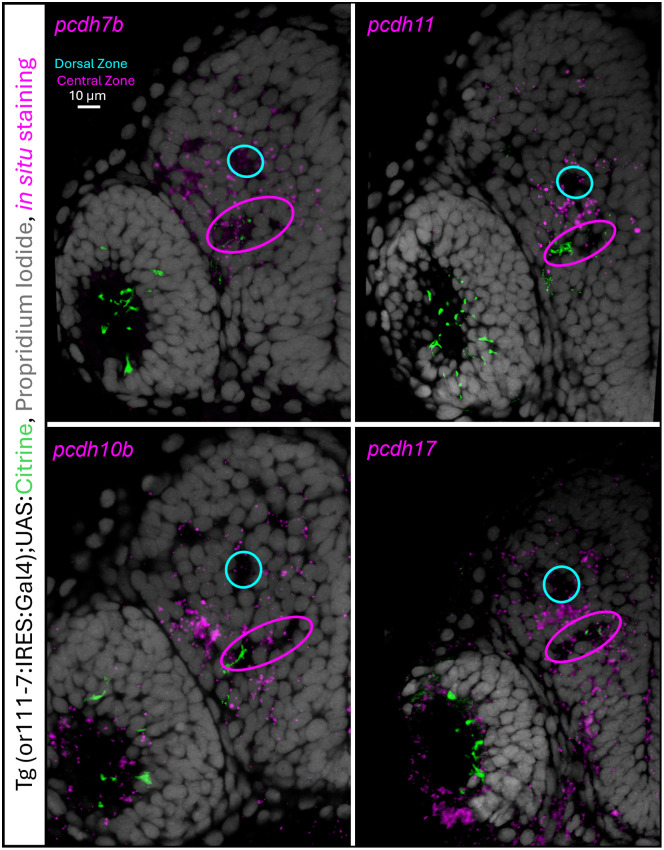
Expression patterns of delta1-protocadherins *pcdh7b* and *pcdh11* and delta2-protocadherins *pcdh10b* and *pcdh17* in the embryonic olfactory bulb. Fluorescent *in situ* hybridization for each protocadherin at 60 hpf. Each image was constructed (see Methods) from the hybridization signals (magenta) of three superimposed preparations overlayed on stained nuclei from one preparation (grey). The dorsal zone (cyan) and central zone (magenta) are denoted by ovals and the scale bar represents 10µm. The embryos originated from crossing two transgenic lines, Tg(or111-7:IRES:Gal4) and Tg(14xUAS:GAP-Citrine), in which CZ projecting OSN axons are labeled with Citrine.

*In situ* hybridization (ISH) was performed to visualize protocadherin expression at 36, 48, and 60 hpf in wholemount embryos as previously described [67]. *Pcdh7b*, *pcdh10b*, and *pcdh11* were expressed at all these times, while *pcdh17* expression was first detected at 48 hpf (S4 Fig). The locations of protocadherin expression were examined in more detail in 60 hpf embryos. Confocal images of fluorescent axons in embryos expressing Tg(*or111–7*:IRES:Gal4, [68] were used to align multiple ISH preparations in three dimensions using IMARIS (see Methods). Each individual image in Fig 10 represents *in situ* signal from three separate sample preparations overlayed onto each other (see Methods). All four protocadherins were expressed in diffusely scattered cells within the olfactory epithelium. This was expected as previous work showed that OSNs expressing ORs from different clades are interspersed amongst each other at these stages of development [13]. *Pcdh7b* is sparsely expressed in cells surrounding the DZ protoglomerulus (Fig 10 top left). *Pcdh11* is expressed in cells surrounding the DZ protoglomerulus with the highest levels of expression in the cells that separate the DZ and CZ protoglomeruli (Fig 10 top right). *Pcdh10b* is most highly expressed in small region dorsal and lateral to the CZ protoglomerulus (Fig 10 bottom left). Finally, *pcdh17* is most highly expressed in surrounding the CZ protoglomerulus. These results show that these four protocadherins are expressed in unique patterns within the olfactory bulb, and therefore, in distinct subsets of olfactory bulb cells.

## Discussion

In this study we explored the molecular differences between Olfactory Sensory Neurons (OSNs) that express main olfactory bulb odorant receptors (ORs) from different OR clades. OSNs expressing clade A or B ORs project axons to the Central Zone (CZ) protoglomerulus, while OSNs expressing clade C ORs project to the Dorsal Zone (DZ) protoglomerulus. We expected to identify guidance-related genes that are differentially expressed between these two groups of sensory neurons.

Un-supervised clustering of single-cell expression profiles was driven by cellular maturity levels more than OSN identity as defined by the predominant OR expressed by an OSN. OSNs are continuously generated in the olfactory epithelium at the developmental stages we surveyed, ORs are expressed very early, and clustering reflected a developmental trajectory containing a variety of differing OSN maturity levels. OR expression was distributed between all OSN maturity levels. OR subfamilies or clades were indiscriminately represented within all UMAP clusters. To detect gene expression differences between OSNs expressing ORs from differing clades, we first categorized cells by the most prominent OR they expressed, grouped OSNs into OR clade A, B, or C expressing categories, and then looked for differences in gene expression between these three groups.

One surprising finding is that relatively few differentially expressed genes were detected in OR clade A as compared to OR clade B expressing OSNs. This held true for candidate guidance related genes, transcription factors, and all other genes. In contrast, more genes were found to be differentially expressed in OR clade C as compared OR clades A or B expressing OSNs. This is consistent with OR clade A and clade B expressing OSNs projecting to the CZ glomerulus, while OR clade C expressing OSNs project to the DZ glomerulus. Generally, many of the differentially expressed genes we found were enriched in OR clade C expressing OSNs as compared to OR clade A or B expressing OSNs. Since the CZ and the DZ protoglomeruli are immediately adjacent to one another, our results invite the speculation that over the course of evolution new guidance related genes have been added to the CZ expression repertoire that redirect axon projections to a new nearby target, the DZ protoglomerulus.

In the resulting list of differentially expressed, candidate guidance-related genes, we focused on a group of four non-clustered delta-protocadherin family members [69]. Delta-protocadherins play an important role in neural development and variants are associated with many human neurodevelopmental disorders [70–72].

*Pcdh11* and *pcdh7b* are members of the delta1 subfamily whose four members each contain 7 extracellular cadherin domains and two conserved intracellular domains. *Pcdh10b* and *pcdh17* are members of the delta2 subfamily whose 5 members each contain 6 extracellular cadherin domains, two conserved intracellular domains like those in delta1 protocadherins, and an additional intracellular domain that is shared amongst the delta2-protocadherins. All the delta-protocadherins are homophilic adhesion molecules [73]. Additionally, delta1-protocadherins can bind weakly with some other delta1 members and delta2-protocadherins can bind weakly with some other delta2 members. We found that knocking down three of the four protocadherins identified in this study, *pcdh7b*, *pcdh11*, and *pcdh17*, clearly affected OSN targeting.

### Delta1-protocadherins are required for DZ protoglomerular targetin

Delta1-protocadherins *pcdh11* and *pcdh7b* are most highly expressed in OSNs targeting the DZ protoglomerulus. Their knockdown induced targeting errors in OSNs that normally terminate in the DZ protoglomerulus, reducing the frequency with which they terminate in the DZ. This effect was specific, as *pcdh11* or *pcdh7b* knockdown did not affect OSN axons targeting the CZ protoglomerulus. Axons targeting the DZ successfully navigated close to the DZ protoglomerulus in knockdowns but failed to fully incorporate within it. They tended to curl away and terminate more ventrally outside the DZ. It therefore seems unlikely that these two delta1-protocadherins play an important role in OSN axon pathfinding. Instead, this phenotype is consistent with delta1-protocadherins specifically stabilizing cell to cell contacts either between sensory axons that densely populate the DZ protoglomerulus, or between sensory axons and olfactory bulb dendrites within the protoglomerulus. This would be in keeping with the known homophilic and weaker heterophilic adhesive interactions between delta1-protocadherin family members [73].

P*cdh7b* and *pcdh11* are expressed in OSNs that project to the DZ protoglomerulus, but also in specific cells within the olfactory bulb. The expression pattern of *pcdh11* is particularly striking as it is most highly expressed in cells surrounding the target location for OSNs that express *pcdh11*, the DZ protoglomerulus. These olfactory bulb cells may be interneurons that contribute dendritic processes to the DZ, but unfortunately, we do not have markers that selectively label olfactory bulb neurons whose dendrites contribute to specific protoglomeruli. It is attractive to speculate that the dendrites of *pcdh11* expressing olfactory bulb neurons in the DZ protoglomerulus help to anchor pcdh11 expressing, DZ projecting OSN axons once they enter the protoglomerular neuropil.

Interestingly, the knockdown effects of *pcdh7b* and *pcdh11* are not additive or synergistic. The simplest interpretation of this observation is that they function in series rather than in parallel. For example, *pcdh11* on one neuronal process might bind to and adhere to *pcdh7b* on another. As *pcdh11* appears to be more highly expressed in the bulb than *pcdh7b*, it is possible that olfactory bulb neuronal dendrites expressing *pcdh11* capture *pcdh7b* expressing OSN axons in the DZ protoglomerulus. However, this would be unexpected as delta-protocadherins bind homophilic rather than heterophilic partners most strongly [73]. Cell adhesion experiments suggest that delta-protocadherins expressed together may modulate each other’s adhesive functions [74], raising the possibility that adhesive driven axon sorting could be disrupted by the loss of either one of two delta-protocadherins that are co-expressed in the same OSN.

### The delta2-protocadherin *pcdh17* is required for CZ protoglomerular targeting

Delta2-protocadherins *pcdh17* and *pcdh10b* are most highly expressed in OSNs targeting the CZ protoglomerulus. Their knockdown induces pathfinding errors in OSNs that normally terminate in the CZ protoglomerulus. This effect is specific, as knockdown did not affect OSN axons targeting the DZ protoglomerulus. CZ projecting OSN axons in *pcdh17* knockdowns are less likely to terminate in the CZ protoglomerulus and more likely to terminate elsewhere. Unlike the errors observed in delta1-protocadherin knockdowns, in which general DZ pathfinding appears to remain intact but incorporation of OSN axons into the DZ is impaired; in *pcdh17* knockdowns many axons lose their way long before they approach the CZ protoglomerulus. They wander to ectopic dorsal and/or posterior locations early in their trajectory. Knockdown of the other delta2-protocadherin subfamily member *pcdh10b* had the weakest phenotype of the four protocadherins we examined. The percentage of or111–7 expressing OSN axons terminating in the CZ protoglomerulus is not significantly reduced in *pcdh10b* knockdowns, but there are modest increases in ectopic ventral misprojections.

The early dispersion of BAC:OR111–7 expressing OSN axons from their normal trajectory and their failure to reach the CZ protoglomerulus in *pcdh17* knockdowns lead us to categorize this protocadherin as playing a role in axon guidance rather than target stabilization. One plausible explanation for the phenotype we observe is that strong *pcdh17* mediated homophilic adhesion helps keep CZ projecting axons fasciculated together on their normal trajectory [70], and that the loss of *pcdh17*-mediated adhesion allows axons to escape this pathway. This would be consistent with the report that *pcdh17* promotes axonal fasciculation of mouse amygdalar axons through the recruitment of actin polymerization regulators in the WAVE complex, and the recruitment of Lamellipodin and Ena/VASP to axon-axon contact sites [72]. A similar mechanism for promoting motility upon contact has been proposed for *pcdh10* [75]. *Pcdh10* has been proposed to participate in glomerular segregation in the mouse [76], a process that occurs after the protoglomerular targeting we are examining in this study. In contrast to the idea that delta2-protocadherins promote the motility of fasciculated axons, Asakawa and Kawakami (2018) proposed that *pcdh17* promotes axonal self-avoidance in zebrafish abducent nerve axons [77]. Our *pcdh17* knockdown phenotype does not match the axonal clumping phenotype they describe and is instead more consistent with *pcdh17* promoting fasciculation. We cannot, however, rule out a repellent interaction between OSN axons and *pcdh17* expressing cells in the olfactory bulb.

Our results suggest differing roles for delta1-protocadherins and delta2-protocadherins in the zebrafish olfactory system. We propose that delta1-protocadherins stabilize OSN axon terminations in the DZ protoglomerulus, while the delta2-protocadherin, *pcdh17,* mediates axon fasciculation and guidance well before OSN axons reach their target CZ protoglomerulus.

## Materials and methods

### Ethics statement

All experiments were conducted with the approval of the University of Pennsylvania Institutional Animal Care and Use Committee (protocol #804895).

### Zebrafish lines

Veterinary care was supervised by University Laboratory Animal Resources (ULAR). The transgenic zebrafish line tg(OMP2k:lyn–mRFP)^rw035a^ (OMP:RFP) used in scRNA-seq experiments were obtained from the Yoshihara Lab [24,57]. Axonal targeting experiments were performed using OR BAC transgenic lines TgBAC(or111–7-IRES:GAL4-VP16,myl7:EGFP^zf1062Tg^) and TgBAC(or130–1-IRES:GAL4-VP16,myl7:EGFP ^zf1063Tg^) crossed to the reporter line Tg(14xUAS:GAP-Citrine^p201Tg^) [13,67]. Axonal tracing in the in situ studies was performed on the progeny of Tg(or111–7:IRES:Gal4) [Tg(or111–7:or111–7-IRES-GAL4)^p202Tg^] crossed with Tg(14xUAS:GAP-Citrine). The *pcdh11* mutant line (sa31987) was obtained from the Zebrafish International Resource Center (ZIRC).

### Collection of OSNs for scRNA-seq

Transgenic OMP:RFP fish were crossed to themselves, and embryos were collected after 30 minutes. Embryos were raised at 28.5°C for both 48hpf and 72hpf experimental timepoints. To obtain OSNs at the equivalent of the 36hpf developmental timepoint, embryos were raised at 28.5°C for 24 hours and then 25°C for 24 hours. Zebrafish were anesthetized with tricaine and sacrificed in ice-cold E3. Olfactory tissue containing OSNs was removed from tricaine treated iced embryos by manual dissection and placed in 67% HEPES Buffered Salt Solution (HBSS) with 1% BSA and stored on ice. Olfactory tissue was collected over the course of 2 hours. After tissue collection, the medium was replaced by FACSmax cell dissociation solution (AMSBIO, AMS.T200100) and incubated at 28°C for 30 minutes. During incubation, the samples were agitated by inversion of the tubes every 5 minutes and by gentle pipetting. Cells were combined in a single 15 ml Falcon tube and pelleted by centrifugation at 400g for 6 minutes at 4C. FACSmax dissociation solution was removed and cells were resuspended in HBSS with 25mM HEPES and 1% BSA. OMP:RFP positive cells were isolated from the cell suspension by FAC sorting at the Penn Cytomics and Cell Sorting Resource Laboratory using stringent gating for both fluorescence and cell size to prevent the collection of doublets. Positive single cells were collected in HEPES buffered HBSS with 1% BSA and stored on ice. Cells were spun down by centrifugation and media was replaced with HBSS immediately prior to 10x Genomics sample processing. A detailed protocol is available at protocols.io: **https://dx.doi.org/10.17504/protocols.io.rm7vze1k5vx1/v1**

### 10x genomics scRNA-seq analysis

10x Genomics scRNA-seq sample processing was performed by the Penn Genomics and Sequencing Core at the University of Pennsylvania using the Chromium Next GEM Single Cell 3′ Kit V3. Sequencing was performed on the NovaSeq 6000 with a target of 60,000 reads per cell for each sample. Demultiplexing and genomic alignment to the zebrafish reference genome GRCz11 was performed using cell Ranger version 3.0.1 with default settings. Initial data analysis was performed using R version 3.6.3 and Seurat version 3.2 with subsequent analysis performed in R version 4.3 and Seurat version 4.3. Differential gene expression testing was performed with DESeq2 [78–80]. All scRNA-seq data is available at the Gene Expression Omnibus accession code GSE291626. The scripts used to analyze the data are available at https://github.com/DanielTBarnes/sc-RNAseq-of-Zebrafish-Olfactory-Sensory-Neurons.

### F0 knockdown experiments

A combination of 3 separately targeted CRISPR-Cas9 RNPs per gene was utilized to generate first generation knockdowns [64]. CRISPR guides were chosen based on their predicted likelihood of inducing a frameshift mutation, predicted CRISPR efficiency, and the likelihood of abrogating gene function. We used CHOPCHOP for crRNA design and CRISPRscan for CRISPR efficiency and frameshift prediction [65,81]. A total of 4–8 CRISPR targets were screened per target gene using headloop PCR and the efficiency of successful guides was ultimately determined using the sequences of PCR products bracketing CRISPR cut sites subjected to analysis by DECODR [64,66]. A combination of 3 CRISPR guides were injected into just fertilized embryos resulting from a cross between either TgBACor111–7:IRES:Gal4 or TgBACor130–1:IRES:Gal4 with Tg(UAS:gap43-citrine). Embryos were raised at 28.5°C until 72hpf and then sacrificed for imaging. The design parameters and sequences of the crRNA used for the knockdown experiments along with the primers used for headloop PCR and DECODR sequencing are all available in S1 File. The F0 knockdown procedure itself does not induce errors in OSN targeting. Successful knockdown of the pigmentation gene *slc24a5* does not induce errors in BAC:OR111–7 or BAC:OR130–1 labeled OSN axon trajectories (S5 Fig).

### F0 double knockdown experiments

Double knockdowns of *pcdh7b* and *pcdh11* were accomplished with an injection mix containing 4 CRISPR guides composed of targets 2 and 3 for *pcdh7b* along with targets 1 and 3 for *pcdh11* (Table 1). Matching single gene knockdowns were also generated using comparable 2-guide mixes containing either targets 2 and 3 for *pcdh7b* or targets 1 and 3 for *pcdh11*. Injections were carried out as outlined above; however, the overall injection volume was slightly increased to ensure the final concentrations of individual CRISPR guides were the same in the 2 as compared to the 3 CRISPR guide mixes. Knockdown of the target genes was assessed after every batch of injections via DECODR [66]. Embryos were raised at 28.5°C until 72hpf and then sacrificed for imaging.

### Immunohistochemistry

Embryos were fixed in 4% PFA in 0.1M phosphate buffer (PBS) overnight at 4°C. A CUBIC-based tissue clearing protocol was then performed with the addition of RedDot2 (Biotium 40061) staining to label nuclei [82]. Briefly, embryos were immersed and gently shaken in CUBIC 1 solution for two hours at room temperature. Following several PBS washes and 1 ten-minute PBS-Triton x-100 wash, the treated embryos were stained with RedDot2 in PBS (1:75) overnight. The embryos were washed several times in PBS and immersed in CUBIC 2 solution until they were imaged. Embryos were mounted and imaged in CUBIC 2 solution.

### Imaging

All images were acquired using a Leica SP5 confocal microscope with a 63x oil immersion lens. Z-stacks were generated with a step size of 1 μm beginning at the posterior end of the olfactory epithelium and proceeding through to the anterior margin of the olfactory bulb. Stacks were generated using FIJI and examined using ClearVolume [83,84].

### Analysis

Image data were anonymized and protoglomerular targeting was assessed independently by two individuals. Axon terminations were scored based on their termination point relative to their normal protoglomerular target. Misprojections on the anterior-posterior, dorsal-ventral, and medial-lateral axes of the fish were quantified. Discrepancies in scoring between the two individual scorers were resolved by consensus and then the data were unblinded. Statistical differences were assessed using a one-tailed Fisher’s exact test in GraphPad Prism version 10.3.0, GraphPad Software, Boston, Massachusetts USA, www.graphpad.com.

### In situ hybridization

Embryos for *in situ* hybridization experiments were generated by crossing transgenic lines Tg(*or111–7*:IRES:Gal4) [Tg(or111–7:or111–7-IRES-GAL4)] and UAS:Citrine [Tg(14xUAS:GAP-Citrine)]. Embryos were collected and raised at 28.5°C. Embryos were sacrificed and fixed in 4% PFA overnight followed by serial dehydration in methanol. Embryos were stored in methanol at -80C.

*In situ* probes were generated for each protocadherin by PCR amplification from cDNA followed by cloning into a Dual-promoter TA cloning kit (Thermo, K207040). The primers used to generate *in situ* hybridization probes are available in S1 File. The delta-protocadherins are non-clustered and have few splice variants. Each protocadherin contains 1 large exon which encompasses the majority of the protocadherin functional domains. Full length (~800 bp) probes were designed to target these exons. Digoxin probes were made using a DIG RNA labeling kit (Sigma-Alderich, 11175025910) followed by LiCl/Ethanol purification. *In situ* hybridization experiments were performed as previously described [85]. Following *in situ* hybridization, citrine expressing OSNs were labeled with anti-GFP (1:100; Rockland Immunochemicals, 600–101–215) and visualized with donkey anti-goat IgG Alexa Fluor 488 secondary antibody (1:500; Invitrogen). Nuclei were stained with propidium iodide (330ug/ml, Sigma-Aldrich, P4170). Fish heads were mounted for imaging in Vectashield Plus antifade medium (Vector Laboratories, H-1000).

To construct the images in Fig 10, z-stacks from three separate wholemount *in situ* preparations were superimposed upon one another using IMARIS 10.0 following protocols previously described [60]. Images were aligned to maximize overlap between transgenically labeled OSNs and individually identifiable protoglomeruli. This involved rotating each preparation in three dimensions to obtain the best match. Once they were aligned, the *in situ* signals were added together in a three-dimensional virtual space. The resulting virtual olfactory bulb was then digitally re-sectioned at 1 micron intervals to generate the final images.

## Supporting information

S1 Fig Uniform Manifold Approximation and Projection visualizations of olfactory sensory neuron maturity markers. UMAPs showing the expression of selected markers of OSN maturity including those represented in the dot plot in Fig 1E.
(TIF)

S2 Fig Uniform Manifold Approximation and Projection visualizations of immune cell related genes. Cluster 7 (bottom left) of the UMAP in Fig 1B was primarily composed of cells expressing immune system related genes.
(TIF)

S3 Fig The trajectories of olfactory sensory neurons in uninjected control and either *pcdh11* or *pcdh17* F0 knockdown embryos. Three dimensional reconstructions of OSN axon trajectories were produced from confocal Z-stacks using ClearVolume. Axonal trajectories of BAC:OR130–1 OSNs in (A) an uninjected control embryo or (B) a *pcdh11* F0 knockdown embryo. Axonal trajectories of BAC:OR111–7 OSNs in (C) an uninjected control embryo or (D) a *pcdh17* F0 knockdown embryo. The locations of the DZ protoglomerulus (blue) and the CZ protoglomerulus (cyan) were identified by the absence of nuclear staining (not included in these images).
(TIF)

S4 Fig The time courses of *pcdh11*, *pcdh7b*, *pcdh17*, and *pcdh10b* expression in the olfactory bulb. *In situ* probes for each protocadherin (magenta). were applied to 36, 48, 60 hpf embryos. The embryos contained a transgene that labels axons projecting to the CZ protoglomerulus. Optical sections are shown from individual preparations that have not been rotated or re-aligned.
(TIF)

S5 Fig Knockdown of a pigmentation gene does not affect OR axon targeting in the olfactory bulb. The solute carrier family 24 member 5 (*slc24a5*) gene is required for normal melanocyte differentiation and migration. Mutations in this gene are associated with reduced pigmentation in zebrafish and humans (Lamason et al, 2005). Its function is not expected to affect the guidance or targeting of olfactory sensory neuron axons. F0 knockdown of *slc24a5* did not increase axon targeting errors of either OR111–7 or OR130–1 expressing OSNs.
(TIF)

S1 File The sequences of CRISPR crRNA, DECODR sequencing primers, headloop primers, and *in situ* hybridization primers for each protocadherin in this study. (XLSX)
